# Changes of AM Fungal Abundance along Environmental Gradients in the Arid and Semi-Arid Grasslands of Northern China

**DOI:** 10.1371/journal.pone.0057593

**Published:** 2013-02-25

**Authors:** Yajun Hu, Matthias C. Rillig, Dan Xiang, Zhipeng Hao, Baodong Chen

**Affiliations:** 1 State Key Laboratory of Urban and Regional Ecology, Research Center for Eco-Environmental Sciences, Chinese Academy of Sciences, Beijing, China; 2 Freie Universität Berlin-Institut für Biologie, Dahlem Center of Plant Sciences, Plant Ecology, Berlin, Germany; 3 Berlin-Brandenburg Institute of Advanced Biodiversity Research (BBIB), Berlin, Germany; Dowling College, United States of America

## Abstract

Arbuscular mycorrhizal (AM) fungi are ubiquitous symbionts of higher plants in terrestrial ecosystems, while the occurrence of the AM symbiosis is influenced by a complex set of abiotic and biotic factors. To reveal the regional distribution pattern of AM fungi as driven by multiple environmental factors, and to understand the ecological importance of AM fungi in natural ecosystems, we conducted a field investigation on AM fungal abundance along environmental gradients in the arid and semi-arid grasslands of northern China. In addition to plant parameters recorded *in situ*, soil samples were collected, and soil chemo-physical and biological parameters were measured in the lab. Statistical analyses were performed to reveal the relative contribution of climatic, edaphic and vegetation factors to AM fungal abundance, especially for extraradical hyphal length density (HLD) in the soil. The results indicated that HLD were positively correlated with mean annual temperature (MAT), soil clay content and soil pH, but negatively correlated with both soil organic carbon (SOC) and soil available N. The multiple regressions and structural equation model showed that MAT was the key positive contributor and soil fertility was the key negative contributor to HLD. Furthermore, both the intraradical AM colonization (IMC) and relative abundance of AM fungi, which was quantified by real-time PCR assay, tended to decrease along the increasing SOC content. With regard to the obvious negative correlation between MAT and SOC in the research area, the positive correlation between MAT and HLD implied that AM fungi could potentially mitigate soil carbon losses especially in infertile soils under global warming. However, direct evidence from long-term experiments is still expected to support the AM fungal contribution to soil carbon pools.

## Introduction

Arbuscular mycorrhizal (AM) fungi are ubiquitous symbionts of higher plants and important components of most terrestrial ecosystems [1]. AM fungi can efficiently take up mineral nutrients, especially phosphate, from soil, and then deliver them to the host plant; in exchange, 4–20% of plant photosynthates are directly transferred from plant to AM fungi to support development of the symbiosis [1]. Therefore, these symbiotic soil fungi are recognized as critical links between the above- and belowground parts of ecosystems [2].

The occurrence of the AM symbiosis is influenced not only by host plant species, but also by the environmental factors, such as temperature [3], pH [4], [5], and soil fertility [6]. The influences of particular environmental factor on AM fungi have been extensively examined by quantifying root colonization, hyphal length density or spore density in soil along environmental gradients at different scales, including aridity [7], salinity [8], nutrient [9], land degradation [10] and temperature [3] gradients. For instance, Heinemeyer and Fitter (2004) demonstrated that higher temperature could stimulate colonization of AM fungi on host plants and development of extraradical mycelium [11]. As commonly recognized, AM fungi facilitate plant acquisition of limited soil resources; AM plants would therefore be more abundant in ecosystems with less available soil nutrients [6]. On the other hand, soil pH was reported to be a key factor influencing the abundance and distribution of soil fungi and bacteria [12]. Positive correlation between soil pH and root colonization by AM fungi was recorded in both acid and alkaline soils under low available phosphate conditions [4], [13]–[14]. However, despite of established relationships between AM fungal occurrence and particular environmental factors, few studies to date have examined the effects of multiple environmental factors on AM fungal abundance in a natural ecosystem.

AM fungi promote the growth of host plants, by means of providing mineral nutrients and water [15], [16] and up-regulating photosynthesis [17]. In this way, AM fungi are also involved in soil carbon cycling, as plant photosynthates are the original source for soil carbon pools, and better plant growth would subsequently lead to more carbon input into soil ecosystem. Moreover, extraradical mycelium (ERM) of AM fungi and their products (including glomalin-related soil protein, GRSP) could potentially stimulate soil aggregation [18], while the soil aggregates would provide protection for organic carbon from rapid degradation by microbes [18]. Several studies revealed that soil warming could promote ERM development [19], [20]. AM fungi are therefore supposed to increase carbon inputs to soil carbon pool under climatic warming. However, Rillig et al. [19] reported that GRSP content and soil aggregate stability decreased under higher temperature, despite of increasing ERM abundance, which would potentially lead to a reduction in soil carbon sequestration. Obviously, more systematic investigation is required to reveal the relationship between AM fungal abundance and soil carbon sequestration along environmental gradients.

In the present study, the arid and semi-arid grasslands of northern China were selected as target research area. We chose this area mainly for two reasons: (1) the area exhibits high variations in multiple environmental factors, including temperature and precipitation; (2) the soils in this area are generally nutrient-poor, therefore the native plants are expected to be highly dependent on mycorrhizal symbioses, and AM fungi thus could play key roles in ecosystem processes. We recorded plant parameters *in situ* and collected soil samples along climatic gradients, and then AM fungal parameters and key soil chemo-physical properties, including SOC and soil particle-size, were analyzed in the lab. Statistical analyses were performed to quantify the relative contribution of climatic, edaphic and vegetation factors to AM fungal abundance, and also to reveal the relationships between AM fungal parameters and SOC across environmental gradients. The study therefore aimed at revealing the regional distribution pattern of AM fungi as driven by multiple environmental factors, and also at uncovering the ecological importance of AM fungi in natural ecosystems

## Materials and Methods

### 2.1. Description of the study area and soil sampling

**Figure 1:**
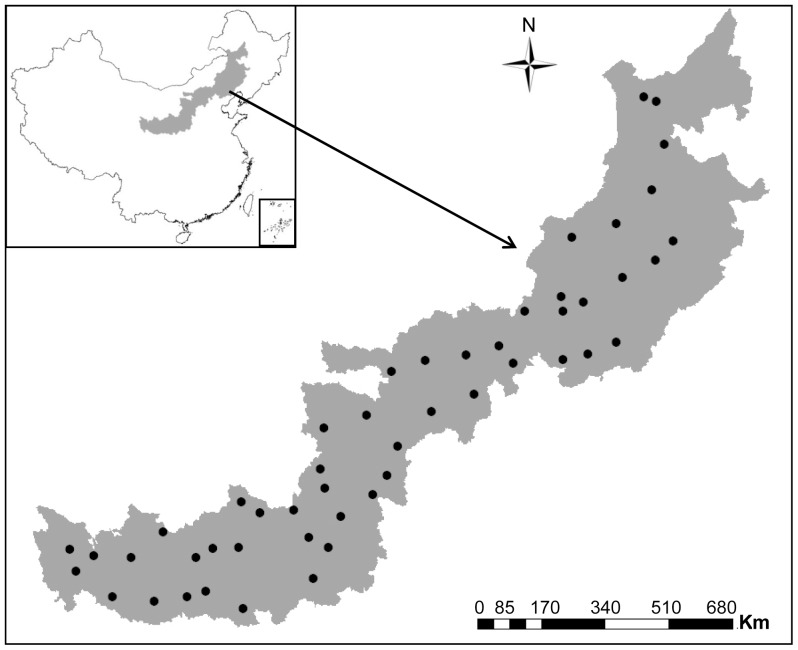
Sampling sites in the arid and semi-arid grasslands of northern China. Data obtained from the National Fundamental Geographic Information System (NFGIS, http://ngcc.sbsm.gov.cn/), maps edited using ArcGIS9.3 (ESRI, Redlands, CA, USA).

The study area represents sylvosteppe, typical steppe and desert steppe from east to west in northern China (Fig. 1). It is situated in the semi-arid and arid zone and covers a total area of 440 000 km^2^. Data of mean annual temperature (MAT) and the mean annual precipitation (MAP) (40 years from 1969 to 2009) were obtained from China Meteorogical Data Sharing Service System (http://cdc.cma.gov.cn/). The climatic data of sampling sites were generated from 64 climatic stations across the study region using the Kriging interpolation. MAT in the study area ranged from 2.8°C to 9.6°C, and MAP ranged from 281 mm to 534 mm. Approximately 80% of the precipitation occurs from May to September, while the growing season extends from late April to October. Vegetation of the region predominantly consists of grasses such as *Leymus chinensis*, *Setaria viridis*, *Tribulus terrestris, Cenchrus incertus*, *Lespedeza davurica*, *Artemisia capillaries*, *Carex humilis*, *Carex onoei* and *Chloris virgata*.

Fifty sampling sites were arranged based on the random stratified design from thirty-one sampling grids, which were generated by the overlap map of temperature and precipitation data in the study area using ArcGIS. Each sampling site was picked based on where the vegetation was minimally disturbed by human activities, such as grazing. Five 1*1 m^2^ (quadrats) within an area of 100 m^2^ were designated at each site for recording plant parameters including plant species and coverage. Fifteen soil cores (3 cm in diameter and 15 cm in depth) were sampled from random locations within the 100 m^2^ sampling plot and thoroughly mixed into one composite soil sample after vegetation cover and litter removal. The field study did not involve any privately-owned land or protected area of land (such as national park), and the sampling did not involve any endangered or protected species. Therefore, no specific permits were required for the described field studies.

The field investigation and soil sampling were performed in August, as most plant species reach the peak biomass during this period of a year. Soil samples were stored in polyethylene bags in a refrigerated box at 4°C. After transportation to the laboratory, the soil samples were passed through 2 mm mesh to remove plant debris, thoroughly homogenized and separated into two subsamples. One subsample was frozen at −80°C for molecular analysis, another was air-dried for analysis of soil chemo-physical properties, extraradical AM hyphae and glomalin-related soil protein fractions. At the same time, fresh roots were manually collected from the soil samples for measuring AM fungal colonization.

### 2.2. Soil physical and chemical properties

Soil pH was measured in a 1∶2.5 (v/v) soil∶water suspension with a digital pH meter (PHS-3C, Shanghai Lida Instrument Company, China). SOC was determined by the Walkley-Black dichromate oxidation procedure [21]. Soil available phosphorus was measured according to the method described by Olsen et al. [22]. Soil available nitrogen was measured by an alkaline hydrolysis method [23]. Soil particle-size was analyzed by a laser diffraction technique using a Longbench Mastersizer 2000 (Malvern Instruments, Malvern, England). Before sample analysis, soil organic matter was destroyed with H_2_O_2_ (30%, w/w) at 72°C and sodium hexametaphosphate, following by sonication for 30 s to disperse aggregates. Three parallel measurements were performed for each soil sample to minimize the experimental errors.

The data of soil particle-size were fractionated into clay (0–2 µm), silt (2–50 µm) and sand (50–2000 µm) according the classification system of U.S. Department of Agriculture (USDA). Soil particle size distribution (PSD) was also characterized by the volume fractal dimension value (D value), which is calculated by using the following formula [24]:
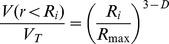
Where *r* represents the particle size, *R_i_* represents the particle size of grade *i* in the particle size grading, *V*(*r<R_i_*) represents the volume of soil particles with a diameter less than *R_i_*, *V_T_* represents the total volume of the soil particles, *R_max_* represents the largest particle diameter of the soil. The soil particle sizes from 0 to 2000 µm were divided into 64 classes using the software package of the laser particle analyzer.

### 2.3. Glomalin-related soil proteins

Two GRSP pools were extracted from soil samples following the modified methods as described by Wright and Upadhyaya [25]. In brief, 0.5 g of soil samples were autoclaved at 121°C for 30 min in 4 ml of 20 mM citrate buffer (adjusted to pH = 7.0) and the collected supernatant was defined as easily extractable GRSP (EE-GRSP). Moreover, 0.5 g of soil samples was extracted at 121°C for 1 h in 4 ml of 50 mM citrate buffer (adjusted to pH = 8.0). The extraction process was repeated three times until the supernatant was almost clear or showing a light yellow color, and the collected supernatant was defined as total GRSP (T-GRSP). The protein concentration was determined by the Bradford assay using bovine serum albumin as standards.

### 2.4. Quantification of AM fungal intra- and extraradical colonization

Subsamples of fresh roots were cleared with 10% KOH and stained with Trypan blue following a modified procedure described by Phillips and Hayman [26], omitting phenol from solutions and HCl from the rinse. Thirty randomly selected 1 cm root segments were examined for intraradical AM colonization at 200× magnification according to Trouvelot et al. [27]. Quantification of extraradical colonization followed the modified membrane filter protocol of Jakobsen et al. [28]. In brief, duplicate 4 g soil sample were blended with 250 ml water and hyphae in 5 ml aliquots were collected on 25 mm membrane filters (1.2 µm pore size) and stained with Trypan blue. Hyphal length was recorded in 25 random fields of view per filter. The length of stained hyphae on the filters was determined by the grid line intercept method at ×200 magnification [29]. Hyphal length of each soil sample was measured with six replicates.

### 2.5. Extraction of soil DNA, primer specificity testing and quantitative PCR analyses

Microbial DNA was extracted from 0.5 g soil, using a Fast DNA SPIN kit for soil samples (Bio 101, La Jolla, Calif.) according to manufacturer's instructions. Real-time PCR assays were conducted on an iQ5 real-time detection system (Bio-Rad Laboratories Hercules, CA). Each 25 µl amplification reaction contained the following reagents: 12.5 µl 2×SYBR Premix Ex Taq (Takara), 1 µl of each primer (5 µmol/µl), 1 µl template DNA (20 ng/µl), and 10.5 µl H_2_O. The primer pair ITS1F(5′-TCCGTAGGTGAACCTGCGG-3′) and 5.8 s (5′-CGCTGCGTTCTTCATCG-3′) was adopted in PCR for all fungal groups, and the PCR procedures were as follows: 30 s at 95°C, followed by 40 cycles at 95°C for 5 s, 30 s at 53°C for annealing, and 72°C for 45 s [30]. The primer pair AMV4.5NF (5′-AAGCTCGTAGTTGAATTTCG-3′) and AMDGR (5′-CCCAACTATCCCTATTAATCAT-3′) was used for PCR amplification of AM fungal group. This primer pair was chosen because of a suitable target sequence (300 bp) and broad amplification spectrum of AM fungi. It was shown by a pyrosequencing approach to amplify the four AM fungal orders of *Archaeosporales*, *Diversisporales*, *Glomales* and *Paraglomerales* from the soil samples [31], [32]. The PCR procedures for AM fungi were as follows: 30 s at 95°C, followed by 40 cycles at 95°C for 5 s, 30 s at 58°C for annealing, and 72°C for 45 s.

To identify specificity of the primer pairs AMV4.5NF/AMDGR for our soil samples, three randomly selected PCR products were selected to construct a mini-library for sequencing. Around 20 randomly selected positive plasmids from each sample were sequenced and then compared with sequences in the NCBI nucleotide database. >70% sequences were identified as the AM fungal taxa in each sample, which confirmed the high specificity of the primer pairs. Amplified products were purified using DNA purification system columns (Promega) and ligated into pGEM-T Easy vector according to the manufacturer's instructions. Positive recombinant bacterial clones were identified by Colony PCR using the vector-specific T7/SP6 primers. Standard curves were generated using a 10-fold serial dilution of the standard plasmid containing the target region amplified by the fungal group and AM fungal primers respectively. A melting curve was performed to confirm the size of specific PCR products. Real-time PCR data collection and the analysis were conducted by iQ5 optical system software v1.0 (Bio-Rad Laboratories, Hercules, CA). The real-time PCR assays were performed in triplicates

### 2.6. Statistical analysis

**Figure 2:**
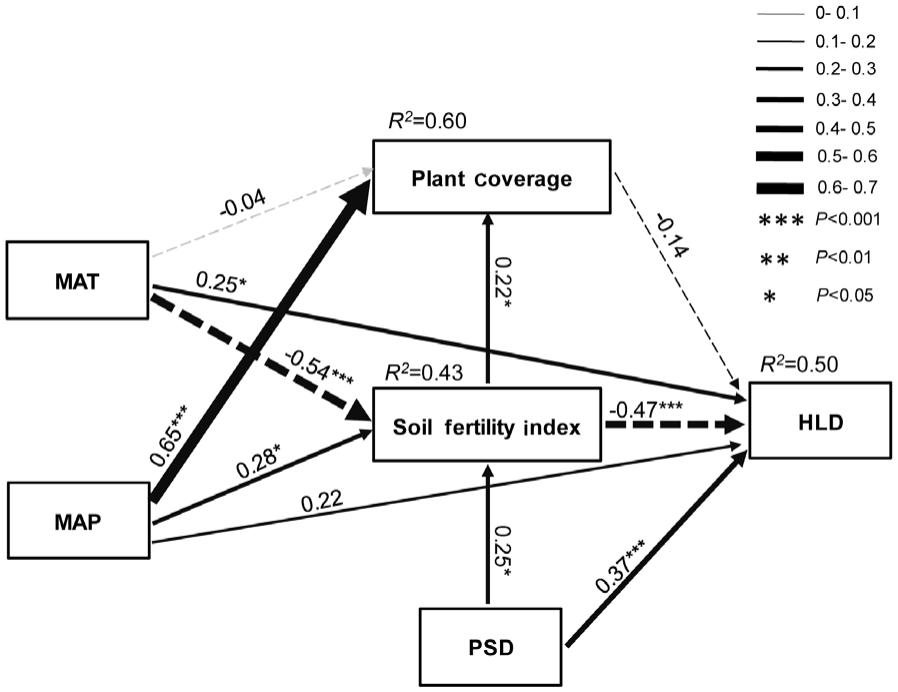
The structural equation model (SEM) showing the hypothesized causal relationships between environmental factors and HLD. Width of arrows indicates the strength of the standardized path coefficient, solid lines indicate positive path coefficients and dashed lines indicate negative path coefficients, *R^2^* values represent the proportion of variance explained for each endogenous variable. ***P<0.001; **P<0.01; * *P*<0.05.

Mean values of the measurements were used for statistical analysis. Simple and multiple linear regression models were employed to evaluate the effects of environmental variables on the HLD of AM fungi. All data for stepwise regression analysis were log(X+1) transformed to meet assumptions of normality, except for soil pH, which was in logarithmic form. Four procedures of stepwise regression were tested against climatic variables, plant variables, soil variables and all variables in combination, respectively. Because of the high collinearity between clay and sand in the soil particle-size data (clay, silt and sand), just clay and silt were picked for the stepwise regression analysis. Pearson correlation analysis was also applied to elucidate the relationships between AM fungal parameters and SOC and MAT, these statistical analyses were performed using the SPSS16.0 software package for windows (version 13.0, SPSS Inc, US). An *a priori* structural equation model (SEM) was applied to test for direct and indirect effects of climate, plant coverage, soil particle size distribution and soil fertility on the HLD according to hypothesized causal relationships (see Fig. 2), using the Amos 17.0 software package (Smallwaters Corporation, Chicago, IL, USA). To simplify the model interpretation, we performed some data reduction by using synthetic variables. Soil fertility index (SFI) and soil particle size distribution (PSD) were used to represent the soil chemo-physical properties. SFI was a synthetic variable derived from the first axis of the principal component analysis (PCA) of soil organic carbon, available phosphorus and available nitrogen; the 99.6% of the variation explained by the first axis in the total suite of soil nutrient (SOC, AN, AP) indicated that this index was a suitable representation of soil fertility. The principal component analysis was performed using CANOCO 4.5 for Windows [33]. *P*-values and χ^2^ values were used to test the structural equation model fit, high *P*-values (*P*>0.05) and small χ^2^ values indicate that the data fit the model well. The goodness-of-fit index (GFI) [34] and the root mean square error of approximation (RMSEA) were also reported considering that the χ^2^ value is usually influenced by sample size. A GFI value higher than 0.9 and a RMSEA value lower than 0.07 suggest that the model shows a significant fit [34].

## Results

### 3.1. Variation of environmental factors and AM fungal parameters

The minimum, maximum, mean and coefficient of variation (CV) of environmental factors (climatic, plant and soil variables) and AM fungal parameters across all soil samples are displayed in Table 1. Most of the climatic, plant and soil variables exhibited high spatial variation in the study area, and the CV values ranked as follows across all examined environmental variables: SOC (86%)>Available N (81%)>Available P (52%)>Plant richness (50%)>Clay (46%)>Silt (41%)>Sand (40%)>Plant coverage (34%)>Plant Simpson index (31%)>MAT (23%)>MAP (12%)>pH (9%).

**Table 1:** Minimum, maximum, means and coefficient of variation (CV) of climatic, edaphic, vegetation and AM fungal parameters in the arid and semi-arid grasslands of northern China (n = 50).

	Variable	Minimum	Maximum	Mean	CV
Climatic	MAT (°C)	2.8	9.6	7.1	0.23
	MAP (mm)	281	534	401	0.12
Edaphic	pH	6.1	10	8.1	0.09
	SOC (mg g^−1^ dry soil)	0.86	46	13	0.86
	Available P (mg kg^−1^ dry soil)	0.87	11	3.1	0.52
	Available N (mg kg^−1^ dry soil)	8.4	259	70	0.81
	Clay (%)	0.02	8.2	3.7	0.46
	Silt (%)	5.4	74	46	0.41
	Sand (%)	18	96	50	0.40
Vegetation	Plant richness	2.2	25	10	0.50
	Plant coverage (%)	0.12	1.0	0.61	0.34
	Plant Simpson index	0.85	3.6	2.2	0.31
AM fungi	Extraradical AM hyphae (m g^−1^ dry soil)	0.75	9.8	3.7	0.58
	Intraradical AM colonization (%)	2.1	68	31	0.52
	Ratio of AMF/Fungi copy number	0.02	0.15	0.06	0.50
	EE-GRSP(g kg^−1^ dry soil)	0.25	1.3	0.75	0.36
	T-GRSP(g kg^−1^ dry soil)	0.30	6.0	2.6	0.58
	EE-GRSP/SOC (%)	0.02	0.37	0.1	0.80
	T-GRSP/SOC (%)	0.12	0.41	0.24	0.29

### 3.2. Effect of environmental characteristics on the extraradical AM hyphae

Both simple and multiple linear regression models were used to assess the relationship between HLD and observed environmental variables. Simple regression showed that HLD increased with increasing MAT, clay content and soil pH, while decreased with increasing SOC and soil available N (Table 2). Stepwise multiple regression model based on all observed environmental variables also identified significant influences of MAT, clay, and soil available N, and among which MAT was the most significant explanatory variable (Table 3).

**Table 2:** Outputs from linear regressions between HLD and environmental parameters.

Dependent variables	Variables	*F* (num df, den df)	Intercept (*P*)	Regression (*P*)	*R^2^*
HLD	MAT	16.26(1, 48)	−0.98(0.417)	**0.67(<0.001)**	0.25
	MAP	0.42(1, 48)	2.01(0.453)	0.004 (0.519)	0.01
	pH	5.17(1, 48)	−3.69(0.266)	**0.92(0.027)**	0.10
	SOC	11.61(1, 48)	4.82(<0.001)	**−0.83(0.001)**	0.19
	Available P	3.33(1, 48)	4.77(<0.001)	−0.34(0.074)	0.06
	Available N	10.11(1, 48)	4.83(<0.001)	**−0.02(0.003)**	0.17
	Clay	6.18(1, 48)	2.19(0.002)	**0.42(0.016)**	0.11
	Silt	1.60(1, 48)	2.77(0.001)	0.02(0.212)	0.03
	Sand	1.89(1, 48)	4.77(<0.001)	−0.02(0.176)	0.04
	Plant richness	3.19(1, 48)	4.81(<0.001)	−0.11(0.080)	0.06
	Plant coverage	1.14(1, 48)	4.66(<0.001)	−1.51(0.291)	0.02
	Plant Simpson index	0.92(1, 48)	4.67(<0.001)	−0.43(0.343)	0.02

Note: Significant regressions (*P*<0.05) are highlighted in bold.

**Table 3:** Outputs from multiple regression analysis of climatic, edaphic and vegetation factors contributing to HLD in the arid and semi-arid grassland of northern China.

Variable	Coefficient	Cumulative *R^2^*	*P*
Climatic factors			
MAT	1.08	0.26	<0.001
Intercept	−0.35		0.14
Edaphic factors			
Clay	0.64	0.11	<0.001
SOC	−0.34	0.38	<0.001
Intercept	0.57		<0.001
Vegetation factors			
NA			
All independent factors			
MAT	0.65	0.26	0.026
Clay	0.42	0.34	0.001
Available N	−0.20	0.40	0.022
Intercept	0.13		0.699

Note: Three independent stepwise models were constructed by restricting to plant variables (plant richness, plant coverage, plant Simpson index), climate variables (MAP, MAT) and soil variables (pH, SOC, available P, available N, clay, silt). The fourth stepwise model introduced all variables (n = 50).

The structural equation model was used to assess the extent of direct and indirect effects of environmental factors on HLD (Fig. 2). The model exhibited a reasonable fit based on our hypothesis (χ^2^ = 3.43, df = 4, *P* = 0.49, GFI = 0.98, RMSEA<0.001) and it could explain 50% of the variance in the biomass of AM fungal mycelium. The causal model showed that HLD increased with increasing MAT and PSD, while it decreased with increasing soil fertility. The path coefficients (λ) for direct and indirect effects on HLD are displayed in Table 4.

**Table 4:** Impact of environmental factors on HLD assessed by structural equation model (SEM) including direct, indirect and total effect coefficients based on hypothesized causal relationships.

	Path coefficient (λ)		
	direct path	indirect path	total effects
MAT	0.25	0.28	0.53
MAP	0.22	−0.23	−0.01
PSD	0.37	−0.12	0.25
Plant coverage	−0.14	0	−0.14
Soil fertility index	−0.47	−0.04	−0.51

### 3.3. Relationships between AM fungal parameters, SOC and MAT

**Figure 3:**
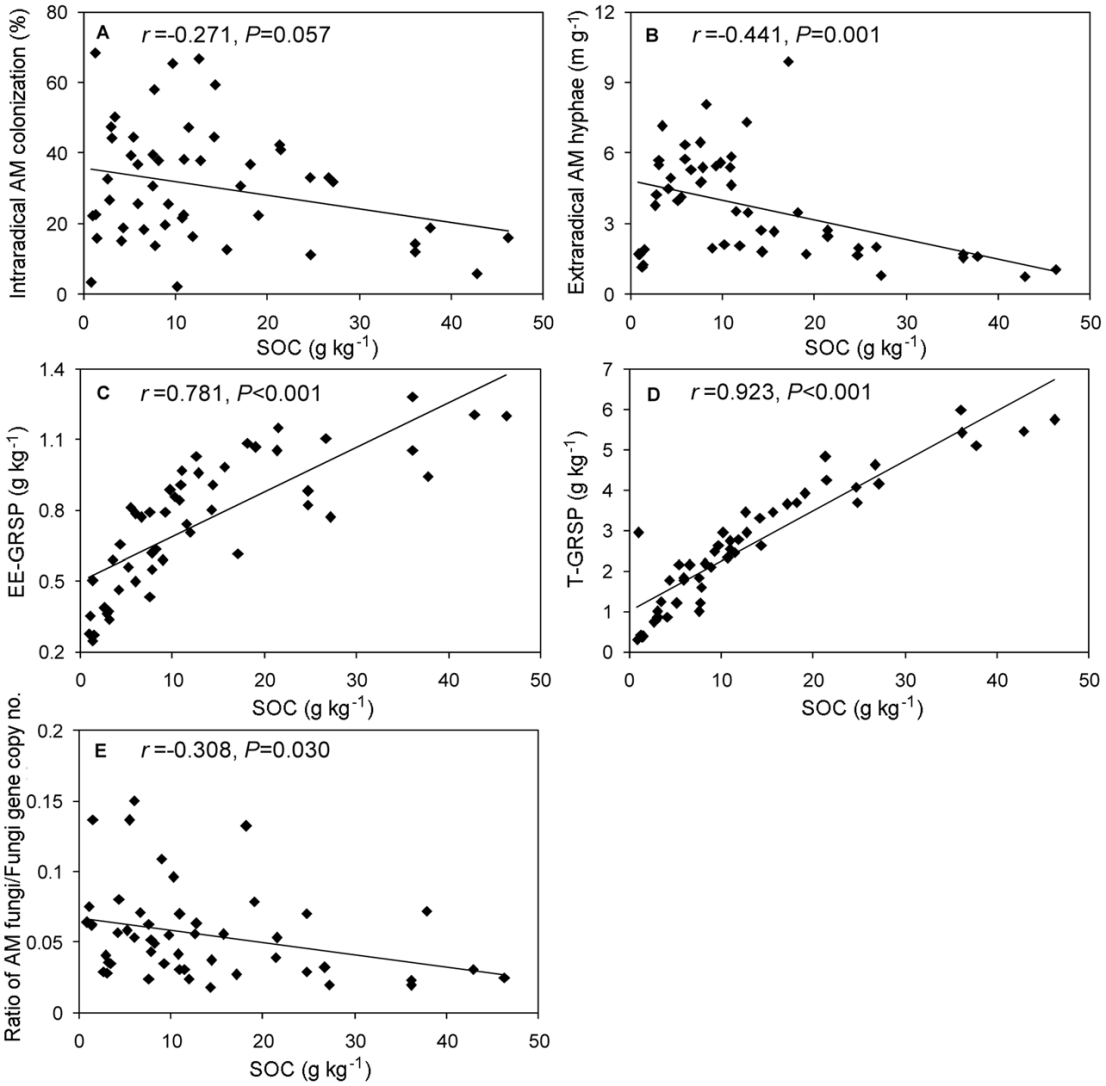
Relationships between AM fungal parameters and SOC. (A) intraradical AM colonization and SOC; (B) extraradical AM hyphae and SOC; (C) EE-GRSP and SOC; (D) T-GRSP and SOC; (E) AMF/fungi gene copy number and SOC.

**Figure 4:**
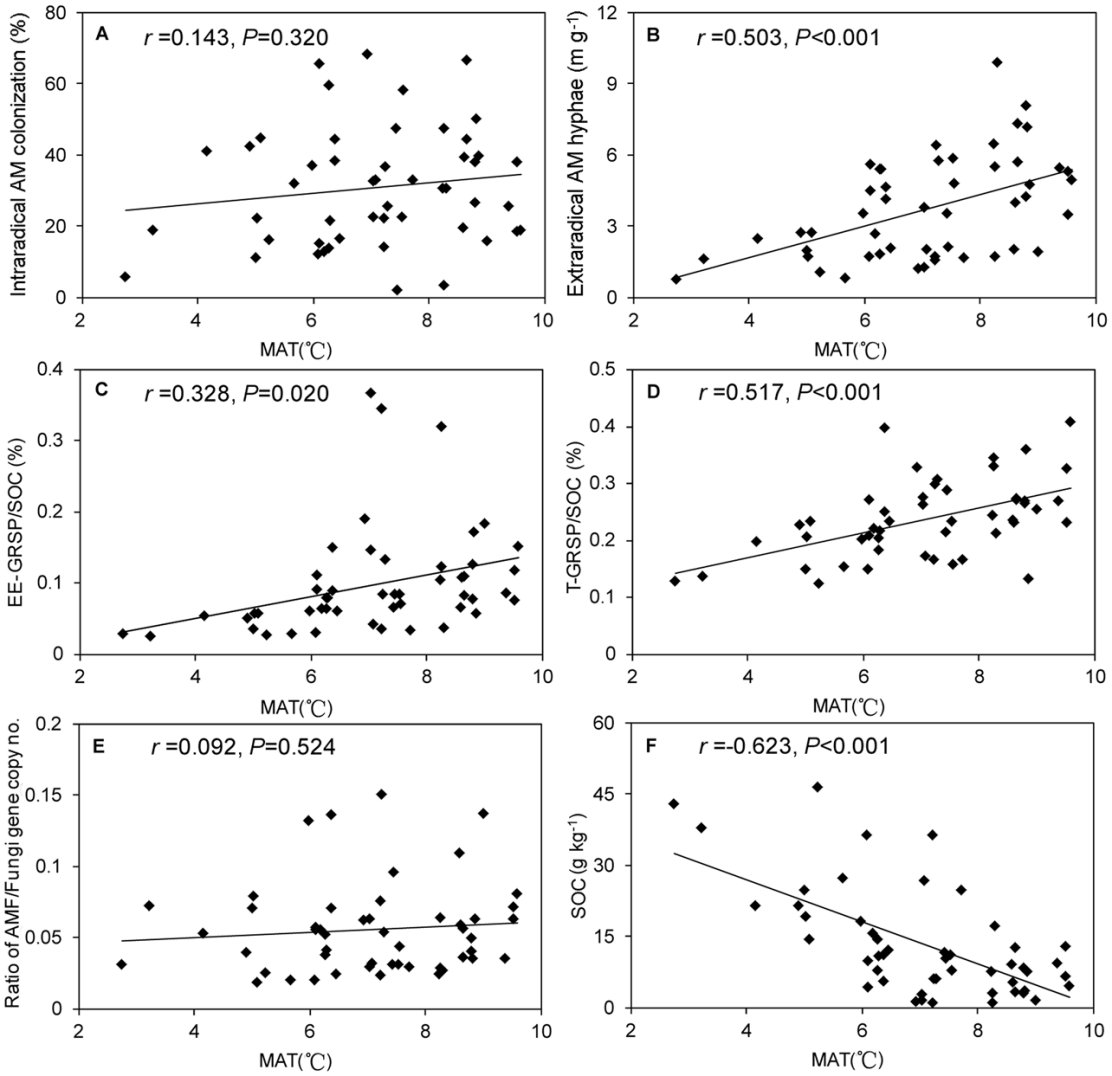
Relationships between AM fungal parameters, SOC and MAT. (A) intraradical AM colonization and MAT; (B) extraradical AM hyphae and MAT; (C) EE-GRSP/SOC and MAT; (D) T-GRSP/SOC and MAT; (E) AMF/fungi gene copy number and MAT; (F) SOC and MAT.

In order to better understand the relationship between AM fungi and soil carbon, a set of AM fungal parameters were examined for their relationship with SOC using Pearson correlations. The results showed that HLD negatively correlated with SOC. Although no significant correlation was found between intraradical AM colonization and SOC, most of the high values of AM colonization appeared in lower SOC soils (Fig. 3A, B).The ratio of the AM fungi to total soil fungi gene copy number was calculated to show the relative abundance of AM fungi using a quantitative PCR assay. The ratios of gene copy number decreased with increasing SOC content (Fig. 3E). However, EE-GRSP and T-GRSP increased with increasing SOC contents (Fig. 3C, D). Although HLD and MAT showed a positive correlation, no significant correlations were found between the intraradical AM colonization, relative abundance of AM fungi and MAT (Fig. 4A, B, E). Both EE-GRSP/SOC and T-GRSP/SOC positively correlated with MAT (Fig. 4C, D). The obvious negative correlation between SOC and MAT was also observed in the study area (Fig. 4F).

## Discussion

Different studies suggested different environmental factors, including live fine root length [35], soil pH [14], nitrogen content [36], or precipitation [37], as main drivers of AM fungal abundance. Similar to a previous investigation [14], in our study significant relationships were also observed between HLD and soil pH, clay content, SOC, available N and MAT, highlighting that likely a complex set of multiple environmental factors affect ERM biomass. Many studies, typically under controlled conditions, demonstrated that higher temperature could stimulate the development of AM fungal mycelium. For example, Gavito et al. (2005) reported that the growth of ERM directly responded to temperature and was independent on the plant roots in a mycorrhizal Ri T-DNA transformed root system [38]. Artificial climate warming study in the field also supported the positive effect of temperature on AM fungal development [19]. In our study, MAT as the key factor positively influenced HLD, suggesting that AM fungi could sensitively respond to climate changes at a regional scale.

The D value, which characterizes soil particle size distribution with higher D value representing lower content of sand [24], significantly affected HLD in this study. It suggested that the soil physical parameter could also stand as a key factor influencing the ERM development. A previous study showed that sandy soil is favorable to AM fungi due to better soil aeration [39], which implies that a negative relationship may exist between D value and HLD. However, a contrary relationship was found in this study. A possible explanation could be that most of the soils sampled from the research area are highly sandy (soil sand content average 50.3%) and could provide well-aerated condition throughout, so that relatively higher sand content did not necessarily lead to better aeration but lower water holding capacity (WHC). As is generally appreciated, water basically drives primary productivity in the arid and semi-arid ecosystems [40]; as a result lower WHC of the soil (higher D value) would be disadvantageous for plant growth and AM fungal development.

Soil nutritional status could stand as another key factor mediating the mutualistic function of mycorrhizal symbioses [6]. In low N or P soils, plant could allocate more carbon to AM fungi and maintain a close symbiotic relationship [41]. On the contrary, in conditions where plants could easily access N and P nutrients in fertile soil, the plant would rely less on the mycorrhizal pathway, and reduce carbon allocation to the symbiosis, as a result, a decline in biomass of AM fungi would be expected [42]. A previous field study carried out in the mesic to semiarid grasslands demonstrated that N fertilization decreased IMC and HLD [36], the meta-analysis also showed that N and P fertilization resulted in decreased mycorrhizal biomass [43]. Similar results obtained in the present study that soil fertility negatively affected HLD provided further evidences to support such a hypothesis. However, some earlier studies showed that N fertilization stimulated AM colonization under P deficiency with the opposite effect under sufficient P supply [36], [44]. It was suggested that the N/P ratio in soil could also mediate the development of mycorrhizal symbioses. In this study, we did not consider the N/P ratio as the soils in this region generally presented N and P co-limited situations and available N and available P were highly correlated (r = 0.643).

As compared to many other studies, we adopted a combination of five AM related parameters to comprehensively assess the relationships between AM fungi and SOC. Considering that saprotrophic fungi act as decomposers in fungal communities [45] while AM fungi are symbiotic and can stimulate soil carbon sequestration [18], higher relative abundance of AM fungi would potentially lead to increasing soil carbon storage via the AM fungi pathway. In this study, quantification of relative abundance of AM fungi by the ratio of gene copy number of AM fungi to total fungi was developed based on real-time PCR assay. A higher relative abundance of AM fungi in the lower SOC soils was observed (Fig. 3E), suggesting AM fungi could play more important role in stimulating soil carbon pools in the infertile than in the fertile soil. However, content of GRSP, which may partially consist of protein secreted by AM fungi [25], decreased with increasing SOC. This was obviously inconsistent with the relationships between other AM parameters and SOC. The possible reason could be that GRSP, unlike the rapidly cycled AM hyphae [46], has relatively slow turnover [47].

Nevertheless, it should be noted that previous studies have reported a positive correlation between abundance of AM fungi and SOC in long-term field experiments [48], [49]. Indeed, a positive relationship is likely to exist between HLD and SOC content across a narrow SOC gradient based on the fact that the AMF can act as a direct contributor to the soil carbon pool. Compared with previous field experiments [48], [49], the inconsistent results in the present study were most likely due to a different spatial scale: the regional scale of the research area provided large variation in the SOC and other environmental factors. To a certain extent, results from the long-term field experiment and our field investigation were incomparable due to different study conditions. Despite all, our data clearly demonstrated that AM fungal abundance increased with the decreasing SOC content at regional scale in the arid and semi-arid grasslands in northern China.

In a previous study, SOC content was found to be negatively correlated with MAT in the low temperature range (MAT≤10°C) by analyzing more than 2000 soil samples obtained from the second soil census in China in the 1980s [50]. The significantly negative correlation between SOC and MAT was also observed in our study. Such results suggested that global warming could possibly lead to a release of soil carbon to the atmosphere by stimulating soil microbial activities and accelerating SOC decomposition [51], [52] However, as AM fungi acquire carbon from the host plant [1] and subsequently redistribute carbon in the mycorrhizosphere, unlike saprotrophic fungi functioning as organic decomposers, could potentially stabilize soil carbon pools under climate warming. Interestingly, a recent study reported that AM fungi increased organic carbon decomposition under elevated CO_2_ by stimulating saprotrophs [53], however, such results did not mean AM fungi could cause soil carbon losses, as the authors examined only freshly added plant residuals in a short-term experiment [54]. Furthermore, the balance of carbon sequestration and decomposition mediated by the interaction of AM with saprotrophic fungi also depends on organic matter quality [54]. Considering that the activity of saprotrophic fungi and bacteria could be quite low in soils with limited carbon resource [55], the relatively higher AM fungal abundance in lower SOC soils implied that they could increase SOC content directly via hyphal turnover and release of GRSP [18]. More importantly, AM fungi could significantly increase the host plant productivity in infertile soil than in the fertile soil [6], this would also largely increase the carbon deposition from plant to soil. With regard to the obvious negative correlation between MAT and SOC in the research area, the increased HLD with the increasing MAT suggested that the AM fungi could be more important to mitigate the losses of soil carbon in the infertile soil under higher temperature.

In conclusion, in the present study we investigated AM fungal abundance at a regional scale in the arid and semi-arid grasslands of northern China. Both climatic and edaphic factors were found to influence AM fungal abundance, while MAT was shown to be the main positive driving factor, and soil fertility the main negative factor for EMH development. The relationships between AM fungal parameters (IMC, HLD, and AM relative fungal abundance), MAT and SOC suggested that AM fungi could potentially play a key role in soil carbon sequestration especially in infertile soils under global warming. It is of critical importance to perform long-term monitoring of AM fungal abundance and SOC to reveal the AM fungal contribution to soil carbon pool under simulated warming conditions.
